# Environmental differences between sites control the diet and nutrition of the carnivorous plant *Drosera rotundifolia*

**DOI:** 10.1007/s11104-017-3484-6

**Published:** 2017-11-18

**Authors:** Joni L. Cook, J. Newton, J. Millett

**Affiliations:** 1 Centre for Hydrological and Ecosystem Science, Department of Geography, Loughborough University, Loughborough, LE11 3TU Leicestershire UK; 2 NERC Life Sciences Mass Spectrometry Facility, Scottish Universities Environmental Research Centre, Rankine Avenue, Scottish Enterprise Technology Park, East Kilbride, G75 0QF UK; 3 Department of Geography, Loughborough University, Loughborough, Leicestershire LE11 3TU UK

**Keywords:** Atmospheric nitrogen deposition, Carnivorous plant, *Drosera rotundifolia*, Ecophysiology, Resource availability, Stable isotopes

## Abstract

**Background and aims:**

Carnivorous plants are sensitive to small changes in resource availability, but few previous studies have examined how differences in nutrient and prey availability affect investment in and the benefit of carnivory. We studied the impact of site-level differences in resource availability on ecophysiological traits of carnivory for *Drosera rotundifolia* L.

**Methods:**

We measured prey availability, investment in carnivory (leaf stickiness), prey capture and diet of plants growing in two bogs with differences in N deposition and plant available N: Cors Fochno (0.62 g m^−2^ yr.^−1^, 353 μg l^−1^), Whixall Moss (1.37 g m^−2^ yr.^−1^, 1505 μg l^−1^). The total N amount per plant and the contributions of prey/root N to the plants’ N budget were calculated using a single isotope natural abundance method.

**Results:**

Plants at Whixall Moss invested less in carnivory, were less likely to capture prey, and were less reliant on prey-derived N (25.5% compared with 49.4%). Actual prey capture did not differ between sites. Diet composition differed – Cors Fochno plants captured 62% greater proportions of Diptera.

**Conclusions:**

Our results show site-level differences in plant diet and nutrition consistent with differences in resource availability. Similarity in actual prey capture may be explained by differences in leaf stickiness and prey abundance.

## Introduction

Carnivorous plants supplement root nutrient uptake by attracting and capturing animal prey, assimilating the nutrients in those prey by digesting and absorbing them, and by using the nutrients for growth and reproduction. The uptake of nutrients through the carnivorous pathway is, therefore, dependent on several linked processes (Juniper et al. 1989). Some of these are obligate (capture, assimilation and use), while others are not (attraction, digestion). The costs involved in carnivory mean that carnivorous plants are predicted to be predominantly restricted to light, wet, low-nutrient environments, where the benefits of carnivory outweigh the costs (Givnish et al. 1984), though significant exceptions exist (e.g. Australian *Drosera* spp. (Pate and Dixon 1982), *Drosophyllum lusitanicum* (Paniw et al. 2017), and most *Byblis* spp. (Conran et al. 2002)). Carnivorous plants can be very sensitive to environmental variability, and can be particularly sensitive to the strength and nature of the plant-soil interaction. Their restriction to nitrogen (N) limited habitats means that root N availability can be an important driver of phenotypic variability (Thorén et al. 2003; Millett et al. 2012), and of population stability (Redbo-Torstensson 1994). Phosphorus (P) can be the limiting macronutrient to for carnivorous plant growth (Ellison 2006), but is far less well-studied. The reliance of carnivorous plants on nutrients from prey varies between and within species. Between-species differences are found due to different trapping mechanisms (Ellison and Gotelli 2001), and due to different life forms (Schulze et al. 1991). Large within-species variations—the reliance on N from prey capture varies between 20 and 60% for *Drosera rotundifolia* (Millett et al. 2012, 2015)—are explained to some extent by differences in root N availability due to the atmospheric deposition of reactive nitrogen (Nr), but other factors, such as prey availability, are also hypothesised to be important (Millett et al. 2015).

In response to differences in root nitrogen availability, carnivorous plants show phenotypic plasticity in several traits relating to the carnivorous habit. For example, the morphology of *Sarracenia purpurea* pitchers—which are modified leaves—changes to being more leaf-like in bogs which receive relatively high levels of Nr deposition (Ellison and Gotelli 2002); the stickiness of ‘flypaper’ leaf-traps of *Drosera rotundifolia* was shown to decrease when root N availability was higher (Thorén et al. 2003). Manipulated addition of prey can lead to increased plant growth and nutritional benefits, for example insect-fed pygmy *Drosera* spp. had threefold greater plant N content and 50% great biomass compared with control (unfed) plants (Karlsson and Pate 1992), insect-fed *Drosophyllum lusitanicum* plants produced greater than fivefold higher dry biomass than control (unfed) plants (Paniw et al. 2017) and insect-fed *Drosera rotundifolia* plants had on average fivefold higher dry weight of summer plants, factor of 1.5 greater number of leaves, factor of 92 greater number of seeds per plant, and had *c*. threefold larger total trapping areas than unfed plants (Thum 1988). Other studies have investigated prey capture (Thum 1989b; Schulze and Schulze 1990; Alcalá and Domínguez 2003) by or diet of carnivorous plants (Lichtner and Williams 1977; Thum 1986; Zamora 1990; Kato et al. 1993; Harms 1999), and shown differences between species where applicable, but there has been limited investigation of how these might impact on plant nutrition. There are, therefore, many studies of environmental impacts on the various components of carnivory in plants, but what has not been investigated is how resource availability influences all stages of carnivory, from investment in carnivory, prey capture and diet, and root vs. prey N nutrition (Millett et al. 2012, 2015).

Factors other than prey availability and root N availability influence investment in and the benefit of the carnivorous habit. Examples are the availability of light and water (Zamora et al. 1998) and competition for resources with other organisms (Brewer 2003; Jennings et al. 2016). For example, leaf stickiness of *Pinguicula vallisneriifolia* plants increased along a light gradient from shade to full sun (Zamora et al. 1998). In terms of plant ecophysiological responses to competition intensity, the allocation of mass by *Sarracenia alata* plants to pitchers (compared with rhizomes) increased by 35% for plants where surrounding vegetation had been removed compared with control plants with vegetation intact (Brewer 2003). For *Drosera capillaris* populations which experience prey niche overlaps with coexisting wolf spiders (*Sossipus floridanus*) and oak toads (*Anaxyrus quercicus*), trap densities of tentacle-like trichomes were greater for plants growing where toads were present and spider webs situated closer to the ground, compared with plants exposed to relatively lower levels of competition intensity (toad absence, webs situated higher above the ground) (Jennings et al. 2016). Thus, in order to complete a comprehensive investigation of the links between different stages of botanical carnivory, the suite of potentially confounding biotic and abiotic factors that influence single or multiple stages of the trait must be considered.

In this study, we measured the obligate components of carnivory (prey capture, and assimilation and use) for the carnivorous plant *Drosera rotundifolia* L. growing in two ombrotrophic (rain-fed) bogs. These sites differed significantly, primarily in the amount of atmospheric Nr deposition and precipitation they receive. This study investigated whether site-level differences in the strength of the plant-soil interaction occurred, and if so, how these impacted on all stages of the trait of botanical carnivory. The objectives focussed on determining whether populations differed in investment in carnivory (trap stickiness), if this led to changes in diet and prey capture rates, and if these then resulted in altered N nutrition (measured using natural abundance stable isotopes). The relationship between prey capture and prey availability was also explored. Specifically, the following hypotheses were tested: 1. investment in carnivory by *D. rotundifolia* (measured as leaf stickiness) would be lower at the high Nr deposition site; 2. as a result of this, prey capture by *D. rotundifolia* would be lower at the high Nr deposition site; 3. diet quality (taxonomic composition and size distribution of captured prey) of *D. rotundifolia* would be similar; and 4. prey N uptake and the reliance of *D. rotundifolia* on carnivory, measured as the percentage contribution of prey-derived N to the plant N budget, would be lower at the high Nr deposition site. The influence of a suite of abiotic factors acting on *D. rotundifolia* plants was investigated, these included light availability to the plants and pore water dissolved inorganic N content and pH.

## Materials and methods

### Study design

Fieldwork was conducted from May to September 2011 in order to measure prey capture and diet of *Drosera rotundifolia* L. plants throughout their active growth season. In order to investigate the influence of resource availability on investment in carnivory, diet and N nutrition of *D. rotundifolia*, two ombrotrophic bogs in the UK were selected. These supported *D. rotundifolia* populations and were in relatively close proximity (*c*. 97 km) but receive different levels of Nr deposition input (Table 1).

**Table 1:** Characteristics of Cors Fochno and Whixall Moss ombrotrophic raised bogs. (a) average site characteristics for 2006 to 2011 (where possible), (b) site characteristics for 2011 during which fieldwork was undertaken, (c) abiotic characteristics of the ten survey plots at each site throughout the 2011 growth season of *Drosera rotundifolia*

(a)							
Site	Location	Mean annual precipitation (mm yr.^−1^)^a^	Mean temperature January / July (°C)^a^	Mean growing season length (d)^b^	Growing season average temperature (°C)^b^	N deposition (g m^−2^ yr.^−1^)^a^	
Cors Fochno	52°30′09 N, 04°00′57 W	1381	3.5/14.7	320	11.6	0.62	
Whixall Moss	52°92′16 N, 02°76′45 W	719	4.0/13.7	296	11.0	1.37	
(b)							
Site	Annual precipitation (mm yr.^−1^)^c^		Temperature January / July (°C)^c^		Growing season length (d)^c,d^	Growing season average temperature (°C)^c,d^	
Cors Fochno	1008		3.7/15.0		324	12.1	
Whixall Moss	565		2.5/14.2		301	11.5	
(c)							
		NH_4_-N (μg l^−1^)	NO_3_-N (μg l^−1^)	NO_2_-N (μg l^−1^)	DIN (μg l^−1^)	pH (pH units)	Light (%)
Cors Fochno	Mean	0	137	711	353	3.4	61
	SE	0	22	5	106	0.05	3.6
Whixall Moss	Mean	468	256	782	1505	3.8	55
	SE	184	67	17	234	0.15	1.7
*t*-test results^e^	*t*	2.539	1.668	3.926	4.481	2.329	−1.499
	*df*	9	11	10	13	11	18
	*P*	0.032	0.124	0.003	0.001	0.040	0.151

NH_4_-N, pore water ammonium content; NO_3_-N, pore water nitrate content; NO_2_-N, pore water nitrite content; pH, pore water pH; light, proportion of ambient light available to survey *Drosera rotundifolia* plants

^a^Data are mean values for 2006–2011 inclusive

^b^Growing season is the number of days with mean temperature ≥ 5 °C. Data are mean values for 2011–2012 inclusive (earlier years unavailable)

^c^Data values for 2011 only. Based on observed meteorological data from automatic weather stations at each site; data accessed from Countryside Council for Wales (Cors Fochno) and Natural England (Whixall Moss)

^d^Growing season is the number of days with mean temperature ≥ 5 °C

^e^Comparing differences between sites

For each site, data were collated on abiotic environmental conditions. Data was used for the year in which this study took place (2011, Table 1b), but also averages for 2006–2011 (Table 1a), which covers the reported potential lifespan of *D. rotundifolia* (around 5 years (Crowder et al. 1990)). Mean annual precipitation and mean January/July temperature were calculated from the E-OBS gridded data set (Haylock et al. 2008) using the KNMI Climate Explorer (van Oldenborgh 1999). Mean growing season length, defined as the number of days with mean temperature ≥ 5 °C, were calculated using data for 2010–2011 inclusive (earlier years unavailable) obtained from automatic weather stations at each site. Atmospheric Nr deposition inputs were estimated using modelled N deposition data provided by a high resolution national model (Smith et al. 2000; NEGTAP 2001). Data are mean values for 2006–2011 inclusive.

Pore water Dissolved Inorganic Nitrogen (DIN) and pH, and the availability of light to *D. rotundifolia* plants were measured at monthly intervals alongside plant measurements and invertebrate sampling (Table 1c). Pore water samples were collected at monthly intervals by lightly squeezing *Sphagnum* and peat at each plot, filtering within six hours of collection using Whatman 0.7 μm GF/F glass fibre micro filters coupled with a sterilised Sterifil aseptic system (Merck Millipore Ltd., UK) and refrigerated prior to analysis for dissolved inorganic nitrogen (DIN = NH_4_^+^-N, NO_3_^−^-N and NO_2_^−^-N) content using ion-exchange chromatography. Mean pore water pH values were calculated as the average of three measurements per plot at each sampling point using a pH probe (Hanna Instruments Ltd., UK). The availability of light to each survey *D. rotundifolia* plant was measured as the proportion (%) of ambient light reaching each plant. The light intensity next to each plant and the ambient light intensity above the vegetation were measured between 10:00 and 14:00 using a handheld light meter (SKP 200 PAR Quantum Sensor, Skye Instruments Ltd., Wales, UK).

Ten plots were selected at each site by randomly selecting areas which contained *D. rotundifolia* plants growing in *Sphagnum*. Fifteen *D. rotundifolia* plants were randomly selected per plot. At four-weekly intervals measurements were made of *D. rotundifolia* plant and leaf traits, and prey capture attributes (Table 2). Leaf area per plant (LA) was calculated by measuring the number of leaves, and the width and length of each leaf (excluding petioles) in-situ. A regression model was used to convert these measurements to actual leaf area. This model was created from image analysis (using ImageJ (Rasband 1997)) of digital scans of randomly selected leaves (*n* = 60, r^2^ = 0.958, *P* < 0.001) (O’Neal et al. 2002) from outside of the plots. Investment in carnivory in terms of the stickiness of the leaves was measured because the ability of a leaf to capture and prevent invertebrates from escaping is dependent on the sticky mucilage secreted at the ends of leaf trichomes (Zamora et al. 1998). Leaf stickiness measurements were taken from 50 randomly selected non-survey *Drosera rotundifolia* plants located in the survey plots during July 2012 due to adverse weather conditions on sampling dates in 2011. An individual, randomly selected leaf from each plant was lightly pressed onto a 5 cm × 1 cm strip of filter paper attached to a handheld universal digital force gauge (Sauter FH2 model, Kern & Sohn, Balingen, Germany), ensuring that all trichomes were adhered to the filter paper. Leaf stickiness was measured as the peak force (N) required to separate each leaf from the filter paper (Thorén et al. 2003).

**Table 2:** Plant and leaf traits of *Drosera rotundifolia* plants growing in two ombrotrophic bogs in the UK. (a) plant attributes, (b) leaf attributes

(a)									
		Mass (mg)	LA (cm^2^)	Leaf mass (mg)	Leaf number (n)	Total N amount (μg N per plant^2^)	Tissue N concentration (μg N mg^−1^ dry mass)	N_dfp_ (μg per plant)	N_dfr_ (μg per plant)
Cors Fochno	Mean	11.89	0.46	5.24	3.87	123.0	10.31	65.7	57.3
	SE	0.78	0.02	0.40	0.12	9.0	0.15	13.6	7.2
Whixall Moss	Mean	17.26	0.68	5.76	4.55	249.8	14.41	69.3	180.5
	SE	1.33	0.04	0.46	0.17	21.7	0.33	15.2	13.2
*t*-test results^1^	*t*	3.477	4.653	0.851	3.354	5.401	11.258	0.176	8.196
	*df*	18	18	18	18	12	18	18	18
	*P*	0.003	<0.001	0.406	0.004	<0.001	<0.001	0.863	<0.001
(b)									
		LA (cm^2^)		Leaf mass (mg)		LMA (mg cm^−2^)	SLA (cm^2^ mg^−1^)	Prey capture (n)	
Cors Fochno	Mean	0.11		3.13		28.70	0.04	1.2	
	SE	0.00		0.17		2.01	0.00	0.1	
Whixall Moss	Mean	0.13		3.96		31.39	0.03	1.1	
	SE	0.00		0.25		1.88	0.00	0.0	
*t*-test results^a^	*t*	2.734		2.733		0.979	−1.192	−1.279	
	*df*	18		18		18	18	10	
	*P*	0.014		0.014		0.341	0.249	0.229	

Mass, total dry mass per plant; LA, leaf area per plant; leaf mass, total leaf dry mass per plant; leaf number, number of live leaves per plant across the 2011 growth season; total N amount per plant (unadjusted for size); tissue N concentration, amount of N per unit plant dry mass; N_dfp_, amount of prey-derived N per plant; N_dfr_, amount of root-derived N per plant

LA, mean area per leaf; leaf mass, mean dry mass per leaf; LMA, leaf mass per area; SLA, specific leaf area; prey capture, number of captured invertebrates per leaf

^a^Comparing differences between sites

At the end of the 2011 growing season (beginning of September), *D. rotundifolia* plants (current year’s growth), a *c*. 10 cm^3^ sample of *Sphagnum* capitula and above-ground material from the co-occurring plants *Eriophorum vaginatum*, *Calluna vulgaris* and *Erica tetralix* from each survey plot were collected, pooled per species per plot and refrigerated prior to preparation for δ^15^N natural abundance stable isotope analysis. *Sphagnum* and non- carnivorous plant samples were analysed for δ^15^N separately. The mean δ^15^N value of *Sphagnum* and *E. vaginatum* was used for the root-derived N end-point of the δ^15^N natural abundance linear mixing model. The δ^15^N values of the mycorrhizal shrubs *C. vulgaris* and *E. tetralix* were excluded from this calculation because significant between-site differences in δ^15^N values for these species were found (but not for *Sphagnum* and *Eriophorum*), which we interpreted as being due to changes in mycorrhizal N uptake in response to the altered N availability and therefore assumed these data to be an inconsistent end-point.

Invertebrate sampling constituted two phases; the first was to determine the diet of *D. rotundifolia* and the second was to determine the δ^15^N of each prey order of the diet. Plant diet throughout the growth season was determined by collecting freshly captured invertebrate prey at four-weekly intervals from ten randomly selected leaves from non-survey *D. rotundifolia* plants within or adjacent to each plot. Invertebrates captured by survey plants were not removed as this would have influenced %N_dfp_ of the plants. The size (length, width) of the leaves from which prey were removed was measured in order to calculate prey capture parameters at a per leaf unit area basis. In order to determine the δ^15^N of each invertebrate order of prey captured by *D. rotundifolia*, samples of background invertebrates were collected at four-weekly intervals throughout the plants’ active growth season in order to obtain sufficient dry mass for δ^15^N stable isotope analysis. As the diet of *D. rotundifolia* is constituted of flying and flightless invertebrates, background invertebrates were sampled by sweep netting and pitfall trapping respectively. As the abundance and relative order abundance of invertebrates varies at different times of day, three sweep net surveys of the vegetation surrounding each plot were conducted at set times (10:00 h, 12:00 h, 14:00 h) for three minutes. Three bait-less pitfall traps, constructed from plastic cups with suspended roofs, were submerged flush with *Sphagnum* capitula at random locations within a 40 cm radius of survey *D. rotundifolia* plants. After each four-week period, the invertebrates caught at each survey method per plot were pooled, counted and stored in saturated NaCl solution prior to preparation for δ^15^N analysis. This preservation method was chosen as it has a lesser alteration effect on δ^15^N and δ^13^C values than other preservation solutions such as formaldehyde and ethanol (Ponsard and Amlou 1999).

Invertebrates were identified to order level, except for Formicidae which were analysed separately as they are typically δ^15^N enriched compared with other Hymenopterans (Schulze et al. 2001). The length of each specimen was measured to 0.1 mm level of precision using a stage micrometer and optical microscope. Background invertebrates outside of the length range of invertebrates captured by *D. rotundifolia* at both sites (0.4–6.5 mm in length) were excluded from the dataset, and remaining background invertebrates pooled per order per plot for δ^15^N stable isotope analysis. δ^15^N and N concentrations of *D. rotundifolia*, neighbouring non-carnivorous plant species and invertebrates were then determined. Plant and invertebrate samples were rinsed with deionised water, dried to a constant weight by placing in a forced-air oven at 70 °C for 72 h and weighed to obtain dry mass measurements. To ensure sample homogeneity, samples were ground to a fine powder by using a pestle and mortar (invertebrates and *D. rotundifolia* samples) or by using a ball mill (Retsch, Germany) (bryophytes and other plant species). Plant and invertebrate material was pre-weighed into 3 × 5 mm tin capsules and analysed for δ^15^N and N concentrations at the NERC Life Science Mass Spectrometry Facility, UK. Nitrogen isotope ratios were analysed by continuous flow using a Thermo Scientific DELTA V Plus isotope ratio mass spectrometer (Thermo Scientific, Germany) interfaced with a Costech ECS 4010 elemental analyser (Costech Instruments, Italy). Three in-house standards (alanine, gelatine and glycine) were run every ten samples for quality assurance. All data are reported with respect to the international standard of AIR (atmospheric N_2_) for δ^15^N (Table 3). Results are reported in δ notation as the deviation from standards in parts per thousand (‰) (Eq. 1), where:

1 $$ {\updelta}^{15}\mathrm{N}=\left[\frac{\frac{15_{\mathrm{N}}}{14_{\mathrm{N}}} sample}{\frac{15_{\mathrm{N}}}{14_{\mathrm{N}}} reference}-1\right]\times 1000 $$

**Table 3:** Mean δ^15^N values per site for the three δ^15^N natural abundance linear mixing model end points used to calculate the relative contribution of prey-derived N to the total N budget of *Drosera rotundifolia* plants: *Drosera rotundifolia* (δ^15^N_*D. rotundifolia*_), co-occurring non-carnivorous vascular plants (*Sphagnum* and *Eriophorum vaginatum*) (δ^15^N_NCVPs_), and weighted invertebrate prey captured by *D. rotundifolia* (δ^15^N_prey_)

		δ^15^N_*D. rotundifolia*_ (‰)	δ^15^N_NCVPs_ (‰)	δ^15^N_prey_ (‰)
Cors Fochno	Mean	−2.47	−5.52	0.08
	SE	0.31	0.45	0.03
Whixall Moss	Mean	−5.08	−7.12	0.07
	SE	0.31	0.57	0.03

Precision was 0.2 ‰ for δ^15^N. The total N content of plant and invertebrate material were also obtained during the δ^15^N analysis.

### Data analysis

The proportions of prey N (%N_dfp_) and root N (%N_dfr_) of the total N budget of *D. rotundifolia* were calculated by incorporating the δ^15^N of *D. rotundifolia*, mean δ^15^N of selected non-carnivorous plants and δ^15^N of weighted invertebrate prey into a single isotope, two end-point linear mixing model (Shearer and Kohl 1989) (Eq. 2).

2 $$ \%{\mathrm{N}}_{\mathrm{dfp}}=\frac{\updelta^{15}{\mathrm{N}}_{\mathrm{A}}-{\updelta}^{15}{\mathrm{N}}_{\mathrm{B}}}{\updelta^{15}{\mathrm{N}}_{\mathrm{C}}-{\updelta}^{15}{\mathrm{N}}_{\mathrm{B}}} $$

where %N_dfp_ represents the relative contribution of invertebrate prey N to the total N budget of *D. rotundifolia* (%), δ^15^N_A_ represents the δ^15^N of *D. rotundifolia*, δ^15^N_B_ represents the mean δ^15^N of *Sphagnum* spp. and *Eriophorum vaginatum* from the corresponding survey plot, and δ^15^N_C_ represents the mean weighted δ^15^N of invertebrate prey captured by *D. rotundifolia*.

This model uses the discrimination in δ^15^N between trophic levels to calculate the proportional contributions of two isotope sources (prey δ^15^N and root δ^15^N) to a single sink (*D. rotundifolia* δ^15^N). We used a weighted δ^15^N value for the prey end-point (Eq. 3). This is a different approach to previous studies of carnivorous plants (Schulze et al. 1991, 1997; Moran et al. 2001; Millett et al. 2003, 2012, 2015), which used a bulk prey sample. The weighted approach should provide a more accurate representation of the prey end-point because insects can vary in their δ^15^N (Vanderklift and Ponsard 2003), and so the composition of carnivorous plant diet could impact on plant δ^15^N even if the amount of N gained from prey does not differ. The weighted mean δ^15^N of invertebrate prey captured by *D. rotundifolia* plants was calculated by incorporating the δ^15^N and percentage N of dry mass of each invertebrate order per survey plot and the proportional dry mass of each order of captured prey to the total dry mass of captured prey per survey plot (Eq. 3):

3 $$ {\updelta}^{15}{\mathrm{N}}_{\mathrm{C}}={\sum}_i^n\left[{aA}_i\times \left(\frac{a{B}_i}{100}\right)\times \left(\frac{a{C}_i}{aD}\right)\right] $$

where δ^15^N_C_ represents the weighted mean δ^15^N of invertebrate prey captured by *D. rotundifolia*, *n* is the total number of orders of captured invertebrate prey at site *a*, *a*A_i_ is the δ^15^N value (‰) of the *i*th invertebrate order per survey plot at site *a*, *a*B_i_ is the percentage N by weight of the *i*th invertebrate order per survey plot at site *a*, *a*C_i_ is the dry mass (mg) of the i*th* invertebrate order per survey plot at site *a*, and *a*D is the total dry mass (mg) of captured invertebrate prey per survey plot at site *a*. %N_dfp_, percentage N in plant dry matter (%N) and the dry mass of *D. rotundifolia* were used to calculate the amount of prey-derived N per plant (N_dfp_). N_dfp_ was also calculated on a per unit dry mass basis.

The total N content of invertebrate prey captured by *D. rotundifolia* plants was calculated by incorporating the percentage N by weight and the dry mass of each prey order captured by the plants per survey plot and the proportional contribution of each prey order to plant diet per survey plot (Eq. 4):

4 $$ {\mathrm{N}}_{\mathrm{C}}={\sum}_i^n\left[\left(\frac{a{B}_i}{100}\right)\times {aC}_i\right] $$

Where *Nc* represents the total N content (mg) of invertebrate prey captured by *D. rotundifolia* plants, *n* is the total number of orders of captured invertebrate prey at site *a*, *a*B_i_ is the percentage N by weight of the *i*th invertebrate order per survey plot at site *a*, *a*C_i_ is the dry mass (mg) of the i*th* invertebrate order per survey plot at site *a*.

To assess the likelihood of *D. rotundifolia* plants to capture invertebrates of a particular order or size class from the background population, the relative index of ‘prey’ capture success (RIPCS) (cf. Zamora 1995) was calculated using count data for each invertebrate order / size class per site (Table 4). To account for potential differences in sampling effort between the background invertebrate sampling methods, RIPCS was calculated individually for each sampling method, as follows (Eq. 5):

5 $$ RIPCS=\frac{C_y}{\sum {B}_{yi}} $$

(*C*, number of invertebrates captured by *D. rotundifolia* in order or size class *y*; Σ*B*_*yi*_, number of background invertebrates sampled by sweep net or pitfall trap in order or size class *y*).

**Table 4:** Relative index of ‘prey’ capture success (RIPCS) values (mean ± 1 SE) for orders (Table a) and size classes (Table b) of invertebrates captured by *Drosera rotundifolia* plants at two ombrotrophic bogs in the UK where RIPCS calculations incorporate potential prey captured by pitfall trap or sweep net

(a)												
		Invertebrate order										
		Acarina	Araneae	Coleoptera	Collembola	Diptera	Formicidae^†^	Hemiptera	Hymenoptera	Lepidoptera	Orthoptera	Site mean^c,f^
Pitfall trap	Cors Fochno^a^	0.28 (0.16)	0.03 (0.01)	0.25 (0.09)	1.11 (0.47)	5.07 (1.45)	0.11 (0.03)	1.13 (0.49)	1.25 (0.39)	1 (0)	0 (0)	1.2 (0.3)
	Whixall Moss^a^	3.13 (1.06)	0.03 (0.01)	0.73 (0.48)	1.55 (0.78)	9 (2.22)	0.13 (0.02)	0.3 (0.08)	1 (0.19)	0 (0)	0.95 (0.28)	1.37 (0.32)
	Overall^b^	1.58 (0.64)	0.03 (0)	0.5 (0.26)	1.35 (0.47)	6.55 (1.28)	0.12 (0.02)	0.64 (0.22)	1.12 (0.2)	0.17 (0.17)	0.85 (0.27)	
Sweep net	Cors Fochno^d^	1 (0)	0.36 (0.12)	1 (0.34)		2 (0.23)	4.8 (1.11)	1.16 (0.27)	2.17 (0.93)	0 (0)		1.61 (0.25)
	Whixall Moss^d^		0.46 (0.11)	0.27 (0.06)	4 (0)	0.79 (0.07)	3.43 (1.14)	0.63 (0.19)	1 (0)	0 (0)		0.97 (0.21)
	Overall^e^		0.41 (0.08)	0.61 (0.18)		1.39 (0.18)	4 (0.8)	0.86 (0.17)	1.58 (0.49)	0 (0)		
(b)												
		Invertebrate size (mm)										
		0.0–0.9	1.0–1.9	2.0–2.9	3.0–3.9	4.0–4.9	5.0–5.9	6.0–6.9	Site mean^i^			
Pitfall trap	Cors Fochno^g^	0.4 (0)	4.2 (1.88)	1.18 (0.28)	0.29 (0.1)	0.01 (0.01)	0.01 (0)	0 (0)	0.94 (0.36)			
	Whixall Moss^g^	4 (0.71)	1.73 (0.76)	0.51 (0.09)	0.11 (0.02)	0.05 (0.02)	0.04 (0.02)	0.01 (0.01)	0.63 (0.18)			
	Overall^h^	3.28 (0.9)	2.97 (1.03)	0.85 (0.16)	0.2 (0.05)	0.03 (0.01)	0.02 (0.01)	0.01 (0.01)	0.78 (0.2)			
Sweep net	Cors Fochno^j^	6.5 (1.75)	3.24 (0.76)	3.09 (1.19)	0.97 (0.31)	0 (0)	0.13 (0.13)	0 (0)	2.46 (0.48)			
	Whixall Moss^j^	2.62 (0.73)	1.39 (0.15)	0.9 (0.08)	1.1 (0.27)	0.38 (0.12)	0.09 (0.07)	0 (0)	0.9 (0.13)			
	Overall^k^	4.69 (1.09)	2.31 (0.43)	2 (0.63)	1.04 (0.2)	0.24 (0.09)	0.1 (0.06)	0 (0)	1.6 (0.24)			

^†^Family level;

^a^LMM (2-way), interaction effect of site x invertebrate order: *F*_(9, 131)_ = 2.229, *p* = 0.024;

^b^LMM (2-way), main effect of invertebrate order: *F*_(9, 131)_ = 17.807, *p* < 0.001;

^c^LMM (2-way), main effect of site: *F*_(1, 131)_ = 2.799, *p* = 0.097;

^d^LMM (2-way), interaction effect of site x invertebrate order: *F*_(6, 82)_ = 0.768, *p* = 0.597;

^e^LMM (2-way), main effect of invertebrate order: *F*_(8, 82)_ = 12.734, *p* < 0.001;

^f^LMM (2-way), main effect of site: *F*_(1, 82)_ = 5.901, *p* = 0.017

^g^LMM (2-way), interaction effect of site x invertebrate size class: F_(6, 111)_ = 1.802, *p* = 0.105;

^h^LMM (2-way), main effect of invertebrate size class: F_(6, 111)_ = 6.894, *p* < 0.001;

^i^LMM (2-way), main effect of site: F_(1, 111)_ = 0.015, *p* = 0.902;

^j^LMM (2-way), interaction effect of site x invertebrate size class: F_(6, 100)_ = 2.492, *p* = 0.027;

^k^LMM (2-way), main effect of invertebrate size class: F_(6, 100)_ = 9.592, *p* < 0.001;

^l^LMM (2-way), main effect of site: F_(1, 100)_ = 7.147, *p* = 0.009

Prey capture by *D. rotundifolia* plants was measured as the mean number of invertebrate prey captured per leaf throughout the plants’ active growth season. We did not count leaves which were unsuccessful at trapping prey as our original aims were focussed on the dietary composition of *D. rotundifolia*. Therefore, our measure of prey capture is an over-estimation. We feel, however, that this still provides a useful measure of differences in prey capture success. The likely bias that this creates will be an under-estimation of differences between the two sites. This is because, if a plant is less effective at catching prey, then it will likely have more unsuccessful leaves, and this difference is absent from our calculations. To quantify the compositional similarity between prey captured by *D. rotundifolia* plants and the background invertebrate communities within and between sites, the Chao-Jaccard abundance-based similarity index (J_Chao_) (Chao et al. 2005) was calculated using summed incidence frequency data from survey plots for each invertebrate order. The J_Chao_ point estimate for each data pairing with 95% confidence intervals derived from 1000 bootstrap replications were calculated using the EstimateS software package (Colwell 2013). To quantify whether the degree of specialisation displayed by *D. rotundifolia* plants differs between sites and with comparison to background invertebrate communities, the probability of an interspecific encounter (PIE) (Hurlbert 1971) was calculated using proportional abundance data per invertebrate order of each sample population. The only exception was the calculation of PIE values for background invertebrate communities at each site, where the weighted proportional abundance data for each invertebrate order was used in order to account for large within- and between-site differences in the total number of invertebrates sampled by sweep net and by pitfall trap.

The data were analysed using ANOVA, univariate GLM, Linear Mixed Models (LMM), independent-samples *t*-tests, linear regression and Pearson’s correlation. Post-hoc comparisons were conducted using Fisher’s Least Significant Difference (LSD) (*P* < 0.05). To test for significant between-site differences in leaf stickiness between *D. rotundifolia* sample populations, univariate GLM was used with stickiness per leaf as the dependent variable and LA per plant as a covariate. ANOVA was used to test for between-site differences in prey capture by *D. rotundifolia* plants. The mean area per *D. rotundifolia* leaf was used as a covariate. To test for the effects of prey characteristics and the interaction effect of site and prey characteristics on RIPCS, the LMM Procedure was used as this enabled Restricted Maximum Likelihood (REML) estimation, which is more suitable for unbalanced data such as these. Residual plots were used to assess for homoscedascity and normal probability plots used to test that data were normally distributed. Normality of residuals was tested using Q-Q plots and histograms of the residuals from each model. Data were log_10_-transformed where appropriate to achieve homoscedascity prior to analysis. All statistical analyses were conducted using IBM SPSS Statistics versions 21 and 22 (IBM, Chicago, USA).

## Results

### Abiotic environment and plant traits

The abiotic environment differed between the two study sites (Table 1). The concentration of DIN in pore water at Whixall Moss was approximately four times that of pore water at Cors Fochno. This is predominantly due to a lack of NH_4_-N at Cors Fochno. pH was low at both sites, but lowest at Cors Fochno. There was no difference between sites in the proportion of incident sunlight intercepted by the vegetation canopy.

**Fig. 1:**
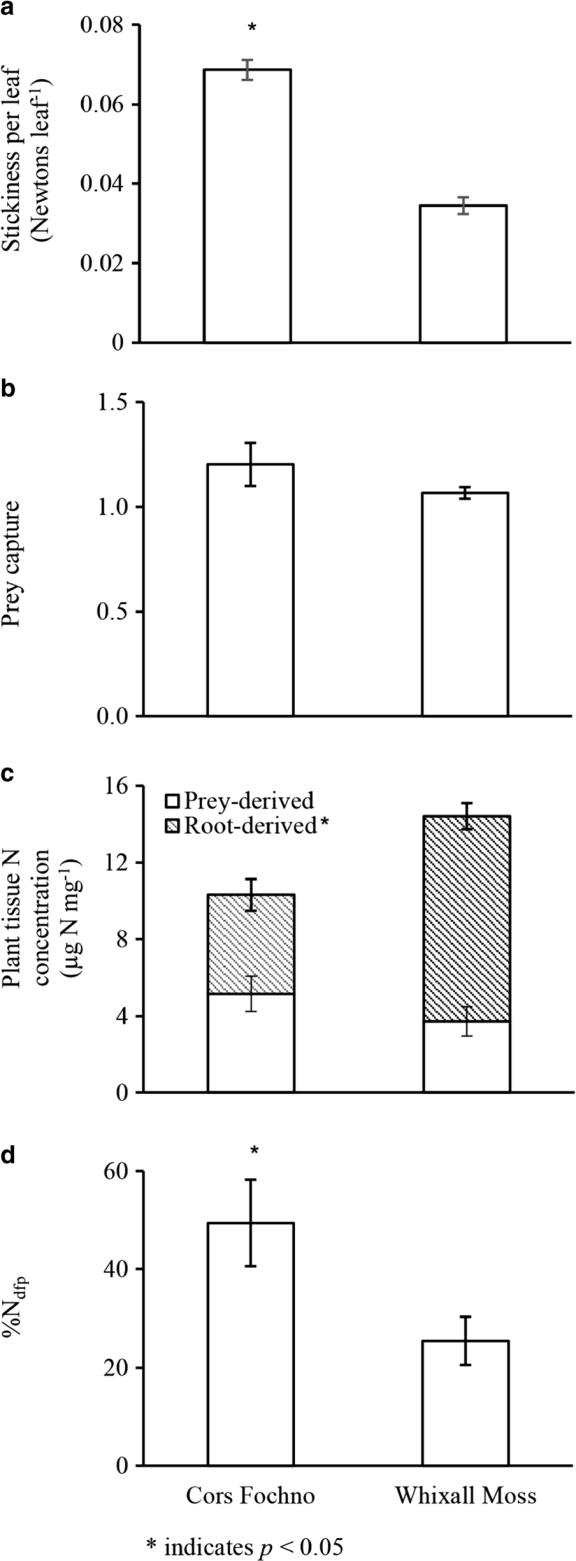
Traits of *Drosera rotundifolia* plants growing in two ombrotrophic bogs in the UK. Presented are the mean ± 1 SE for: (**a**) stickiness per leaf, (**b**) prey capture (number of prey per leaf), (**c**) plant tissue N concentration, and (**d**) percentage contribution of prey-derived N to the total plant N (%N_dfp_)

There were also significant between-site differences in the traits of *D. rotundifolia* plants. On average, dry mass of plants at Whixall Moss was 45% higher, and these plants possessed *c.* 18% more leaves than plants at Cors Fochno (Table 2a). Leaf traits also differed. Plant leaves at Whixall Moss were larger—with higher mass and area—but did not differ in Leaf Mass per Area (LMA) and Specific Leaf Area (SLA), and were about 50% less sticky (univariate ANOVA, *F*_(1,19)_ = 48.356, *p* < 0.001) (Fig. 1a, Table 2b).

For this study, only leaves containing captured prey were selected. For these leaves, there was no statistically significant difference in prey capture between sites. (Fig. 1b; Table 2b). There were, however, significantly greater abundances of background invertebrates sampled by sweep net (representing the dominant prey type of *D. rotundifolia*) at Whixall Moss (mean ± 1 SE = 36.40 ± 1.89) than at Cors Fochno (19.10 ± 1.05; *t*_(18)_ = 7.996, *p* < 0.001). As a result, when the rates of prey capture for all prey taxonomic orders were compared against prey availability, the differences in prey capture (relative index of ‘prey’ capture success, RIPCS) were larger overall at Cors Fochno (RIPCS (mean ± 1 SE) = 1.92 ± 0.17, Whixall Moss = 0.89 ± 0.04, *t*_(10)_ = −6.079, *p* < 0.001).

### Plant nutrition

Plant nutrition also differed between the sites. Plants at Whixall Moss contained over twice the total N amount per plant and possessed higher tissue N concentrations (Fig. 1c; Table 2a). These differences were mainly due to considerably higher total N amounts and concentrations of root-derived N at Whixall Moss (Table 2a). This, along with reduced—but not statistically significant—prey N uptake at Whixall Moss (Table 2a), resulted in a large and significant difference in the proportional contribution of prey N to the total N contained in *D. rotundifolia* plants (Fig. 1d, *t*_(18)_ = −2.375, *p* = 0.029).

### Prey nutrition

The total N content of invertebrate prey captured by plants at Cors Fochno (prey N content (mg): mean ± 1 SE = 1.034 ± 0.456) mean was nearly twofold greater than that of prey captured by plants at Whixall Moss (0.570 ± 0.044; *t*_(18)_ = −7.295, *p* < 0.001).

### Invertebrate communities

**Fig. 2:**
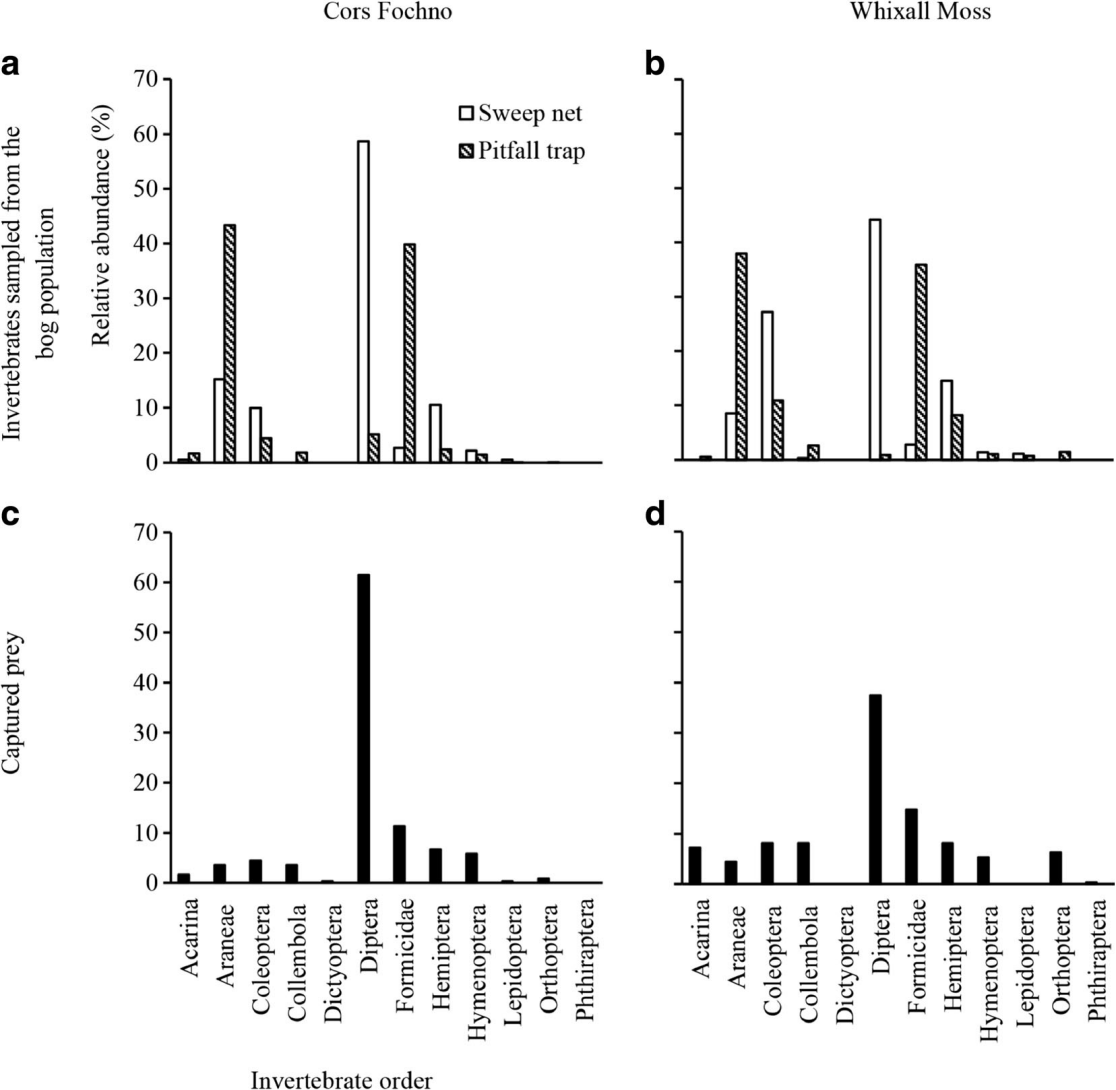
Order distributions of invertebrate taxa sampled using sweep net and pitfall trap (**a**, **b**) and prey removed from *Drosera rotundifolia* plants (**c**, **d**) at two ombrotrophic bogs in the UK. Presented are the relative abundance of each invertebrate order

**Fig. 3:**
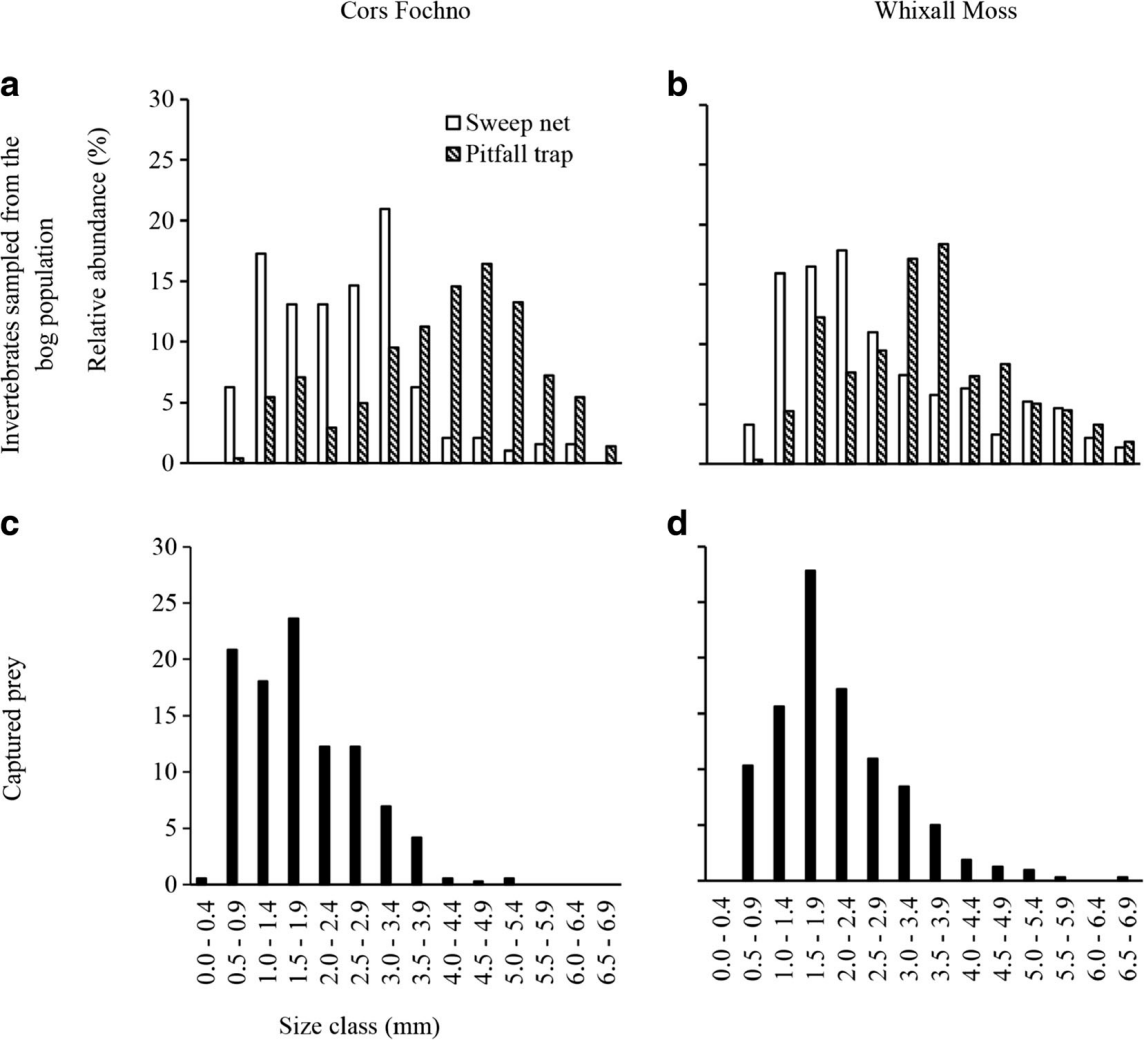
Size distributions of invertebrate taxa sampled using sweep net and pitfall trap (**a**, **b**) and prey removed from *Drosera rotundifolia* plants (**c**, **d**) at two ombrotrophic bogs in the UK. Presented are the relative abundance of each invertebrate size class

Invertebrate populations were composed primarily of Diptera, Formicidae and Araneae (Fig. 2a, b) and did not differ significantly between sites (Chao-Jaccard similarity index (J_Chao_) = 1, 95% CI = 1–1, where a value of 1 indicates no compositional difference; for probability of an interspecific encounter (PIE) data, 95% CIs overlapped at each site (Cors Fochno: PIE = 0.747, 95% CI = 0.703–0.791; Whixall Moss: 0.795, 0.751–0.839; univariate ANOVA, *F*_(1,79)_ = 34.076, *P* < 0.001)). Sweep net and pitfall traps caught different community components at each site. Sweep net samples (hereafter named ‘sweep net invertebrates’) comprised significantly higher proportions of Diptera (mean ± 1 SE = 0.520 ± 0.030) than invertebrates sampled by pitfall trapping (hereafter named ‘pitfall invertebrates’) (0.034 ± 0.009; univariate ANOVA, *F*_(1,39)_ = 346.148, *p* < 0.001; Fig. 2a,b). Pitfall invertebrates constituted predominantly flightless species, with Araneae of the highest relative abundance (RA) (Cors Fochno, RA = 38.0%; Whixall Moss: 43.3%; Fig. 2a,b). Pitfall invertebrates were also larger (mean ± 1 SE = 3.80 ± 0.10 mm), on average, than sweep net invertebrates (2.67 ± 0.07 mm; univariate ANOVA, *F*_(1,39)_ = 128.084, *p* < 0.001; Fig. 3a,b). In sweep net samples, larger proportions of Diptera were present at Cors Fochno (mean ± 1 SE = 0.60 ± 0.03; Whixall Moss: 0.44 ± 0.04; *t*_(18)_ = −3.047, *p* = 0.007).

Overall, the mean invertebrate size did not differ between sites (Cors Fochno: invertebrate length (mean ± 1 SE) = 3.32 ± 0.19 mm; Whixall Moss: 3.15 ± 0.10 mm; univariate ANOVA, *F*_(1,39)_ = 2.736, *P* = 0.107). This result was reflected in the sweep net samples (Cors Fochno: invertebrate length (mean ± 1 SE) = 2.53 ± 0.08 mm; Whixall Moss: 2.81 ± 0.09 mm; univariate ANOVA, *F*_(1,39)_ = 19.988, *P* < 0.001), but not in the pitfall samples (Cors Fochno: 4.11 ± 0.12 mm; Whixall Moss: 3.49 ± 0.10 mm; *F*_(1,39)_ = 19.988, *P* < 0.001).

### Prey capture by *Drosera rotundifolia*

Prey captured by *D. rotundifolia* was composed predominantly of Diptera, (50%) (Fig. 2c,d), followed by Formicidae (13%). Upon comparison of prey with communities sampled by sweep net and pitfall trap, differences in the order composition and size distribution were found (J_Chao_ = 0.99, 95% CI = 0.98–0.99). Specifically, plants captured higher proportions of Diptera than were present in the background populations (*F*_(3,79)_ = 5.231, *p* = 0.003). The proportions of captured Diptera did not, however, differ from communities that were sampled by sweep netting only (*t*_(38)_ = −0.711, *p* = 0.482). The mean length (± 1 SE) of captured prey (1.99 ± 0.06 mm) was smaller than that of the background populations (3.24 ± 0.11 mm; *t*_(55)_ = −10.031, *p* < 0.001).

The composition of captured prey varied between sites (J_Chao_ = 0.96, 95% CI = 0.94–0.98). Plants at Cors Fochno captured an almost twofold greater proportion of Diptera than plants at Whixall Moss (Fig. 2c,d; *t*_(18)_ = −6.823, *p* < 0.001). RIPCS considers prey capture in the context of prey availability. When compared against pitfall invertebrates RIPCS was greatest for Diptera. However, plants at Whixall Moss were about twofold more likely to capture Diptera than plants at Cors Fochno (Table 4a). For sweep net invertebrates, RIPCS was highest for Formicidae and Collembola (Table 4a).

When the data were split into invertebrate size classes the results were similarly consistent. Plants at Cors Fochno were about two times more likely to capture invertebrates in the 0.0–0.9 mm size class than plants at Whixall Moss. This interaction effect of site and invertebrate size class was only significant when RIPCS data were calculated using sweep net invertebrates (Table 4b). Overall, the likelihood of prey capture by plants at Whixall Moss decreased consistently with increasing size class from 0.0–6.9 mm. For RIPCS data calculated using pitfall invertebrates, the interaction effect of site and invertebrate size class failed to reach statistical significance (Table 4b). We suggest the reason for this result is because the between-site differences in counts for each size class were smaller on average for pitfall invertebrates.

No between-site difference in the size of actual prey captured by *D. rotundifolia* was found (*t*_(18)_ = 1.844, *p* = 0.082).

## Discussion

Plant traits that enable efficient acquisition, use and retention of nutrients in low nutrient soils/substrates are considered to be more important than those that enable fast growth rates and high tissue turnover rates (Aerts 1999). Phenotypic variation in the expression of these traits might provide some flexibility and so confer resilience to changes in soil/substrate nutrient availability (Sultan 2000). Understanding how these life history traits might vary in response to changes in soil/substrate nutrient availability, therefore, helps us to understand the extent to which plant communities are resilient to the impacts of atmospheric nitrogen deposition. Botanical carnivory is one such trait which provides a source of nutrients supplemental to those obtained via root uptake. We show, for the first time, the influence of between-site differences in resource availability on all obligatory stages of botanical carnivory—investment in carnivory, prey capture and diet, and nutrition. These differences seem likely to be linked to the impact of atmospheric nitrogen deposition on soil/substrate nitrogen availability and so carnivory, modified by differences in prey availability. Substrate ammonium (NH_4_-N) content differed by the largest magnitude between sites, thus providing evidence of the impact of this soil parameter at the plant ecophysiological level that underpins broader impacts at the species and community levels (Berendse et al. 2001).

We predicted that between-site differences in root N availability would result in differences in root N uptake and prey N uptake, due to differences in trap stickiness, and that this would result in differences in the importance of prey N for the N nutrition of the plants. As has been found in previous studies (Millett et al. 2012, 2015), we found clear evidence that N deposition is a key controlling factor in the N nutrition of *Drosera rotundifolia*. Plants at the high N deposition site were more N replete than plants at the low N deposition site. This was a result of higher tissue concentrations and plant content of root derived N, presumably a consequence of the much higher pore water N concentrations. However, only partial support was present for our fourth hypothesis relating to prey N uptake and plant reliance on carnivory. Plant reliance on carnivory differed between sites—with reliance decreasing with increasing Nr deposition inputs—patterns of prey N uptake, however, did not completely follow our prediction. Prey capture and the amount of N derived from this prey was only marginally higher at the low N deposition site. This was very surprising, given the large differences in pore water N and plant nutrient status, and is in contrary to our predictions (Ellison 2006; Millett et al. 2012, 2015). These results do, however, correspond with several previous findings.

Millett et al. (2015) found a non-linear impact of Nr deposition on prey-derived N among 16 European bogs. The concentration of prey-derived N in plant tissues tended to be higher for plants growing on bogs which received low levels of Nr deposition. For these sites, however, there was a large amount of unexplained between-site variability in tissue concentrations of prey-derived N. Prey availability was speculated to be responsible, and we provide evidence of the potential for this. In the present study, the key reason for the lack of difference in prey capture and prey N uptake appears to be because the abundance of available prey—taken here as being sweep net invertebrates, which better represent the captured prey—at the low N deposition site was lower than that at the high N deposition site. Our results support our first hypothesis; we found that plants did invest more in carnivory—their leaves were stickier—and that they were more successful at catching the potential prey that were available—the RIPCS was higher. These ecophysiological responses enabled rates of prey capture to be maintained at a similar level to the plants at the high N deposition bog, despite the lower prey availability. The lack of statistically significant difference in actual prey capture by plants between sites provide evidence against our second hypothesis and highlights the impact of prey availability on prey capture by *D. rotundifolia*. These results show that the plants are responding as would be expected by evolutionary and ecological theory (Givnish et al. 1984; Thorén et al. 2003), by investing more in prey capture when root N availability is lower. Similar results are reported for other functional types of carnivorous plant; the pitcher:phyllode growth allocation ratio of *Sarracenia purpurea* decreases with increasing root N availability (Ellison and Gotelli 2002). These results also provide evidence that the N nutrition of *D. rotundifolia* is controlled by both abiotic and biotic factors. The ecological consequence of this is not, however, apparent if only plant nutrient contents are measured.

**Fig. 4:**
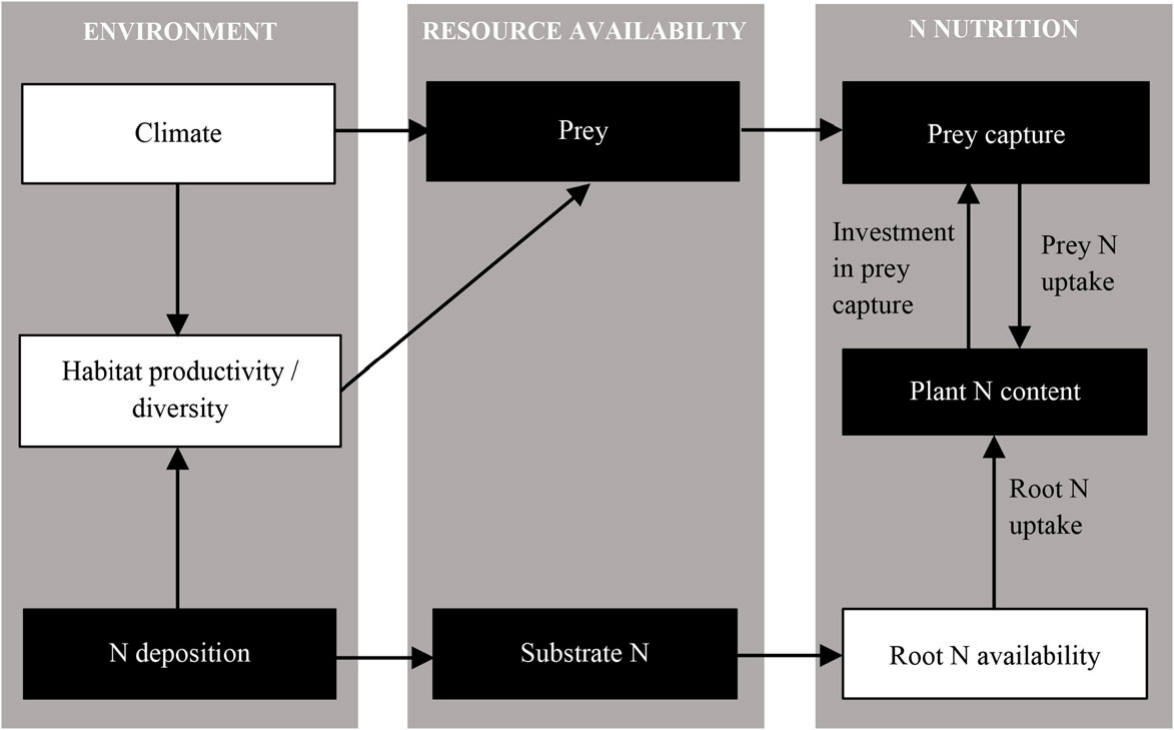
Framework for considering impacts of environmental variability, particularly N deposition, on carnivorous plant nutrition. Presented are the links between environmental variability, resource availability and N nutrition, considering impacts on root N availability and prey availability. Black boxes show the processes considered in the present study

The impact of Nr deposition can be complex, particularly where Nr deposition impacts in different ways on interacting processes (Tylianakis et al. 2008; Bobbink et al. 2010). This can result in non-linear responses to Nr deposition (Payne et al. 2013). The N nutrition of carnivorous plants is a relatively simple example of these more complex interactions. Zamora (1995) demonstrated that prey capture rates were non-linear along an environmental gradient (high light/low moisture – low light/high moisture) because of contrasting patterns of prey availability and investment in carnivory. We show that similar mechanisms might operate in response to Nr deposition. We present a simple framework for considering these interacting impacts (Fig. 4).

Insect abundance is positively related to habitat productivity, which is positively related to Nr deposition (Haddad et al. 2000). Nr deposition also increases root N availability and therefore root N uptake (shown here and by Millett et al. 2012, 2015). Prey capture is a function of prey availability (demonstrated by Alcalá and Domínguez 2003), prey attraction, prey size, and prey escape rates (Gibson 1991) and plant investment in carnivory (shown by Zamora 1990; Zamora et al. 1998), which is itself affected by plant N status (shown here and by Ellison and Gotelli 2002 and Thorén et al. 2003, though the potential importance of other mineral nutrients, particularly P must be considered) and the availability of light (Zamora 1995; Zamora et al. 1998; Alcalá and Domínguez 2003; Thorén et al. 2003) and water (Alcalá and Domínguez 2003). Abiotic factors, such as climate, can also directly impact on insect abundance (Bale et al. 2002). In this study, substantial differences in precipitation inputs between sites may have influenced plant investment in carnivory (for example, by mucilage loss/dilution following rain events), prey availability and retention of captured prey on leaves during digestion. Indeed, results showing similar rates of prey capture by plants at both sites but greater plant investment in leaf stickiness at Cors Fochno may reflect increased investment in prey retention by plants at this site, possibly as a response to the higher precipitation inputs. Feedback between prey availability and investment in carnivory has been shown by *Drosera capillaris* (Jennings et al. 2016), in response to competition for prey with spiders and toads. Prey availability is also linked to the probability of flowering (Krowiak et al. 2017), the production of leaves and seeds (Thum 1988, 1989b), and the retention of prey by carnivorous plants can be impacted by kleptoparasitism (Thum 1989a). Differences in soil N content can also exert potentially interactive effects on plant density and competition (Bobbink et al. 1998). We acknowledge that densities of *D. rotundifolia* plants and co-existing plant species were not measured in this study. The result of this is potentially complex, non-linear responses of plant nutrition to Nr deposition. This framework also demonstrates the potential for other biotic and abiotic factors to affect carnivorous plant nutrition. The key point is that within- and between-site variability in prey availability, and the drivers of this variability, need to be incorporated into consideration of the ecology and evolution of carnivorous plants. Because of this complexity, there is the need for further research utilising *D. rotundifolia* populations across a wide range of sites situated along an atmospheric N deposition gradient.

Overall, our third hypothesis is supported—plant diet was composed predominantly of Diptera and the proportions of Diptera captured by plants did not differ from those present in background sweep net invertebrates. The former results are in alignment with the majority of previous studies exploring the dietary composition of *Drosera* species (Crowder et al. 1990; Ellison and Gotelli 2009; Volkova et al. 2010); the latter show that invertebrates sampled by sweep net, these being predominantly flying species, are more representative of invertebrates captured by *D. rotundifolia* than invertebrates sampled by pitfall trap (predominantly flightless species), and suggest that *D. rotundifolia* utilises a passive trapping mechanism for prey capture. Despite the compositional similarities between populations of captured prey and invertebrates sampled by sweep net, the contribution of flightless species of prey to *D. rotundifolia* diet, as shown by our results, justifies the sampling of potential prey by pitfall trap in addition to by sweep net in order to sample as much of the potential prey spectra as possible.

The choice of sampling method for invertebrate communities affected the calculation of capture likelihood of a specific invertebrate order from the invertebrate communities. For the RIPCS data calculated using sweep net invertebrates, the interaction effect of site and invertebrate order did not reach statistical significance. We suggest that the reason for this result is because there were larger between-site differences in abundances between taxa for pitfall invertebrates than for sweep net invertebrates (e.g. for Diptera, between-site abundances varied by a factor of five when sampled by pitfall trap, but varied only by a factor of 1.5 when sampled by sweep net). The greater capture likelihood of Diptera by plants at Whixall Moss and of invertebrates in the 0.0–0.9 mm size class by plants at Cors Fochno shows that N deposition impacts plant diet in terms of prey taxon and size. Thus, soil N content also impacts on invertebrate community structure through changes in the dietary composition of *D. rotundifolia*. These dietary changes may be simply explained as consequences of changes in the morphology, shape and size of passive traps (Ellison and Gotelli 2009). Similarly, a weak but positive relationship between trap length and prey length is reported for the active traps of *Dionaea muscipula* (Hutchens and Luken 2009). However further research is required to identify the underlying mechanism(s) responsible for the predominant capture of Diptera by *D. rotundifolia* and the relationships between leaf traits, prey availability and plant diet. The twofold greater total N content of prey captured by plants at Cors Fochno compared with plants at Whixall Moss corresponds with between-site differences in %N_dfp_ of *D. rotundifolia*, albeit it is acknowledged that prey N uptake efficiency of *D. rotundifolia*, which itself may be influenced by environmental factors such as temperature and precipitation (Hanslin and Karlsson 1996), was not quantified in this study.

The main limitation of this study is that the use and comparison of only two sites was undertaken. Therefore, we are confident from our results of the differences in plant and invertebrate measures between sites, but we are less able to determine the generality of these differences or the mechanisms driving them. By limiting our study to two contrasting sites we were able to investigate the mechanisms of botanical carnivory in some detail, and importantly in-situ. It would be very difficult to perform a manipulative study because we lack any understanding of the scales at which Nr deposition may impact on insect communities and so on prey availability. To fully understand plant carnivory requires realistic in-situ studies. The results of this study therefore call for further studies that sample a range of sites, but the scale of such a study would be quite large.

The potential for soil nutrient status to influence prey availability and identity, and so prey nutrient acquisition provides an interesting insight into a potential mechanism underlying the evolutionary diversification of leaf morphology and growth forms in carnivorous plants. Growth forms and the morphology of leaf structures in carnivorous plant lineages are highly variable (Gibson and Waller 2009). In the *Drosera* genus, for example, leaf shape varies from flat rosetted (e.g. *Drosera rotundifolia*), spatulate (e.g. *D. spatulata*), peltate (e.g. *D. peltata*) to elongated and almost erect (e.g. *D. intermedia*) (Thum 1986; Albert et al. 1992). Leaf morphology reflects investment in prey capture and influences the amount and type of prey captured; elongated leaves typically capture higher proportions of winged insects than flat rosette leaves (Thum 1986). The differences in prey communities might result in variation in evolutionary cost-benefit balance for different trap morphologies, and so act as an evolutionary driver to trap morphology. We did not measure trap morphology, but this might be an interesting focus for future studies. Growth forms of *Drosera* spp. are also highly diverse, varying from rosette (e.g. *D. rotundifolia*), vine (climbing) (e.g. *D. pallida*) to erect (self-supporting) forms (e.g. *D. stolonifera*) (Schulze et al. 1991; Ellison and Gotelli 2001). As plant reliance on prey-derived N varies between different *Drosera* growth forms (rosette forms are less reliant on %N_dfp_ than erect and vine forms) (Schulze et al. 1991), there is potential for soil N availability to have driven the evolutionary diversification of growth forms through its influence on prey availability and/or identity and/or competition intensity from co-occurring plant species. Future research would benefit from exploring these potential mechanisms across a variety of carnivorous plant genera from different evolutionary lineages.

## References

[CR1] Aerts R (1999). Interspecific competition in natural plant communities: mechanisms, trade-offs and plant-soil feedbacks. J Exp Bot.

[CR2] Albert VA, Williams SE, Chase MW (1992). Carnivorous plants: phylogeny and structural evolution. Science.

[CR3] Alcalá RE, Domínguez CA (2003). Patterns of prey capture and prey availability among populations of the carnivorous plant *Pinguicula moranensis* (Lentibulariaceae) along an environmental gradient. Am J Bot.

[CR4] Bale JS, Masters GJ, Hodkinson ID, Awmack C, Bezemer TM, Brown VK, Butterfield J, Buse A, Coulson JC, Farrar J, Good JEG, Harrington R, Hartley S, Jones TH, Lindroth RL, Press MC, Symrnioudis I, Watt AD, Whittaker JB (2002). Herbivory in global climate change research: direct effects of rising temperature on insect herbivores. Glob Chang Biol.

[CR5] Berendse F, Van Breemen N, Rydin H, Buttler A, Heijmans M, Hoosbeek MR, Lee JA, Mitchell E, Saarinen T, Vasander H, Wallén B (2001). Raised atmospheric CO_2_ levels and increased N deposition cause shifts in plant species composition and production in *Sphagnum* bogs. Glob Chang Biol.

[CR6] Bobbink R, Hornung M, Roelofs JGM (1998). The effects of air-borne nitrogen pollutants on species diversity in natural and semi-natural European vegetation. J Ecol.

[CR7] Bobbink R, Hicks K, Galloway J, Spranger T, Alkemade R, Ashmore M, Bustamante M, Cinderby S, Davidson E, Dentener F, Emmett B, Erisman J-W, Fenn M, Gilliam F, Nordin A, Pardo L, De Vries W (2010). Global assessment of nitrogen deposition effects on terrestrial plant diversity: a synthesis. Ecol Appl.

[CR8] Brewer J (2003). Why don’t carnivorous pitcher plants compete with non-carnivorous plants for nutrients?. Ecology.

[CR9] Chao A, Chazdon RL, Colwell RK, Shen TJ (2005). A new statistical approach for assessing similarity of species composition with incidence and abundance data. Ecol Lett.

[CR10] Colwell RK (2013) EstimateS: statistical estimation of species richness and shared species from samples. Version 9.1.0. [WWW document] URL http://viceroy.eeb.uconn.edu/estimates/. Accessed 4 June 2016

[CR11] Conran JG, Lowrie A, Moyle-Croft J (2002). A revision of *Byblis* (Byblidaceae) in south-western Australia. Nuytsia.

[CR12] Crowder AA, Pearson MC, Grubb PJ, Langlois PH (1990). *Drosera* L.. J Ecol.

[CR13] Ellison AM (2006). Nutrient limitation and stoichiometry of carnivorous plants. Plant Biol.

[CR14] Ellison AM, Gotelli NJ (2001). Evolutionary ecology of carnivorous plants. Trends Ecol Evol.

[CR15] Ellison AM, Gotelli NJ (2002). Nitrogen availability alters the expression of carnivory in the northern pitcher plant, *Sarracenia purpurea*. Proc Natl Acad Sci.

[CR16] Ellison AM, Gotelli NJ (2009). Energetics and the evolution of carnivorous plants – Darwin’s ‘most wonderful plants in the world’. J Exp Bot.

[CR17] Gibson TC (1991). Differential escape of insects from carnivorous plant traps. Am Midl Nat.

[CR18] Gibson TC, Waller DM (2009). Evolving Darwin’s ‘most wonderful plant: ecological steps to a snap-trap. New Phytol.

[CR19] Givnish TJ, Burkhardt EL, Happel RE, Weintraub JD (1984). Carnivory in the bromeliad *Brocchinia reducta*, with a cost/benefit model for the general restriction of carnivorous plants to sunny, moist, nutrient-poor habitats. Am Nat.

[CR20] Haddad NM, Haarstad J, Tilman D (2000). The effects of long-term nitrogen loading on grassland insect communities. Oecologia.

[CR21] Hanslin HM, Karlsson PS (1996). Nitrogen uptake from prey and substrate as affected by prey capture level and plant reproductive status in four carnivorous plant species. Oecologia.

[CR22] Harms S (1999). Prey selection in three species of the carnivorous aquatic plant *Utricularia* (bladderwort). Arch Hydrobiol.

[CR23] Haylock MR, Hofstra N, Klein Tank AMG, Klok EJ, Jones PD, New M (2008). A European daily high-resolution gridded dataset of surface temperature and precipitation for 1950-2006. J Geophys Res Atmos.

[CR24] Hurlbert SH (1971). The non-concept of species diversity: a critique and alternative parameters. Ecology.

[CR25] Hutchens JJ, Luken JO (2009). Prey capture in the Venus flytrap: collection or selection?. Botany.

[CR26] Jennings DE, Krupa JJ, Rohr JR (2016). Foraging modality and plasticity in foraging traits determine the strength of competitive interactions among carnivorous plants, spiders and toads. J Anim Ecol.

[CR27] Juniper BE, Robins RJ, Joel DM (1989). The carnivorous plants.

[CR28] Karlsson PS, Pate JS (1992). Contrasting effects of supplementary feeding of insects or mineral nutrients on the growth and nitrogen and phosphorous economy of pygmy species of *Drosera*. Oecologia.

[CR29] Kato M, Hotta M, Tamin R, Itino T (1993). Inter- and intra-specific variation in prey assemblages and inhabitant communities in *Nepenthes* pitchers in Sumatra. Tropical Zoology.

[CR30] Krowiak A, Herren CM, Webert KC, Einarsson Á, Hoekman D, Jackson RD, Ives AR (2017). Resource gradients and the distribution and flowering of butterwort, a carnivorous plant. Ann Zool Fenn.

[CR31] Lichtner FT, Williams SE (1977). Prey capture and factors controlling trap narrowing in *Dionaea* (Droseraceae). Am J Bot.

[CR32] Millett J, Jones RI, Waldron S (2003). The contribution of insect prey to the total nitrogen content of sundews (*Drosera* spp.) determined *in situ* by stable isotope analysis. New Phytol.

[CR33] Millett J, Svensson BM, Rydin H (2012). Reliance on prey-derived nitrogen by the carnivorous plant *Drosera rotundifolia* decreases with increasing nitrogen deposition. New Phytol.

[CR34] Millett J, Foot GW, Svensson BM (2015). Nitrogen deposition and prey nitrogen uptake control the nutrition of the carnivorous plant *Drosera rotundifolia*. Sci Total Environ.

[CR35] Moran JA, Merbach MA, Livingston NJ, Clarke CM, Booth WE (2001). Termite prey specialization in the pitcher plant *Nepenthes albomarginata* – evidence from stable isotope analysis. Ann Bot.

[CR36] National Expert Group on Transboundary Air Pollution (NEGTAP) (2001) Transboundary air pollution: acidification, eutrophication and ground-level ozone in the UK. Centre for Ecology and Hydrology, Edinburgh

[CR37] O’Neal ME, Landis DA, Isaacs R (2002). An inexpensive, accurate method for measuring leaf area and defoliation through digital image analysis. J Econ Entomol.

[CR38] Paniw M, Gil-Cabeza E, Ojeda F (2017) Plant carnivory beyond bogs: reliance on prey feeding in *Drosophyllum lusitanicum* (Drosophyllaceae) in dry Mediterranean heathland habitats. Ann Bot mcw247. doi:10.1093/aob/mcw24 10.1093/aob/mcw247 PMC5604584 28065921

[CR39] Pate JD, Dixon KW (1982). Tuberous, cormous and bulbous plants: biology of an adaptive strategy in Western Australia.

[CR40] Payne RJ, Dise NB, Stevens CJ, Gowing DJ, Partners BEGIN (2013). Impact of nitrogen deposition at the species level. Proc Natl Acad Sci.

[CR41] Ponsard S, Amlou M (1999). Effects of several preservation methods on the isotopic content of *Drosophila* samples. C R Acad Sci III-Vie.

[CR42] Rasband WS (1997) ImageJ. U.S. National Institutes of Health, Bethesda, Maryland, USA. http://imagej.nih.gov/ij/. Accessed 5 June 2016

[CR43] Redbo-Torstensson P (1994). The demographic consequences of nitrogen fertilization of a population of sundew, *Drosera rotundifolia*. Acta Bot Neerl.

[CR44] Schulze W, Schulze E-D (1990). Insect capture and growth of the insectivorous *Drosera rotundifolia* L. Oecologia.

[CR45] Schulze E-D, Gebauer G, Schulze W, Pate JS (1991). The utilization of nitrogen from insect capture by different growth forms of *Drosera* from Southwest Australia. Oecologia.

[CR46] Schulze W, Schulze ED, Pate JS, Gillison AN (1997). The nitrogen supply from soils and insects during growth of the pitcher plants *Nepenthes mirabilis*, *Cephalotus follicularis* and *Darlingtonia californica*. Oecologia.

[CR47] Schulze W, Schulze ED, Schulze I, Oren R (2001). Quantification of insect nitrogen utilization by the venus fly trap *Dionaea muscipula* catching prey with highly variable isotope signatures. J Exp Bot.

[CR48] Shearer G, Kohl DH, Rundel PW, Ehleringer JR, Nagy KA (1989). Estimates of N_2_ fixation in ecosystems: the need for and basis of the ^15^N natural abundance method. Stable isotopes in ecological research.

[CR49] Smith RI, Fowler D, Sutton MA, Flechard C, Coyle M (2000). Regional estimation of pollutant gas dry deposition in the UK: model description, sensitivity analyses and outputs. Atmos Environ.

[CR50] Sultan SE (2000). Phenotypic plasticity for plant development, function and life history. Trends Plant Sci.

[CR51] Thorén LM, Tuomi J, Kämäräinen T, Laine K (2003). Resource availability affects investment in carnivory in *Drosera rotundifolia*. New Phytol.

[CR52] Thum M (1986). Segregation of habitat and prey in two sympatric carnivorous plant species, *Drosera rotundifolia* and *Drosera intermedia*. Oecologia.

[CR53] Thum M (1988). The significance of carnivory for the fitness of *Drosera* in its natural habitat. 1. The reactions of *Drosera intermedia* and *D. rotundifolia* to supplementary feeding. Oecologia.

[CR54] Thum M (1989). The significance of opportunistic predators for the sympatric carnivorous plant species Drosera Intermedia and Drosera Rotundifolia. Oecologia.

[CR55] Thum M (1989). The significance of carnivory for the fitness of *Drosera* in its natural habitat. 2. The amount of captured prey and its effect on *Drosera intermedia* and *Drosera rotundifolia*. Oecologia.

[CR56] Tylianakis JM, Didham RK, Bascompte J, Wardle DA (2008). Global change and species interactions in terrestrial ecosystems. Ecol Lett.

[CR57] Van Oldenborgh GJ (1999) KNMI Climate Explorer. Koninklijk Nederlands Meteorologisch Institut. http://climexp.knmi.nl. Accessed 8 Sept 2014

[CR58] Vanderklift MA, Ponsard S (2003). Sources of variation in consumer-diet δ^15^N enrichment: a meta-analysis. Oecologia.

[CR59] Volkova PA, Sukhov ND, Petrov PN (2010). Three carnivorous plant species (*Drosera* spp.) in European Russia: peaceful coexistence?. Nord J Bot.

[CR60] Zamora R (1990). The feeding ecology of a carnivorous plant (*Pinguicula nevadense*): prey analysis and capture constraints. Oecologia.

[CR61] Zamora R (1995). The trapping success of a carnivorous plant, *Pinguicula vallisneriifolia*: the cumulative effects of availability, attraction, retention and robbery of prey. Oikos.

[CR62] Zamora R, Gómez JM, Hódar JA (1998). Fitness responses of a carnivorous plant in contrasting ecological scenarios. Ecology.

